# Natural Compounds for Alzheimer’s Disease Therapy: A Systematic Review of Preclinical and Clinical Studies

**DOI:** 10.3390/ijms20092313

**Published:** 2019-05-10

**Authors:** Stephanie Andrade, Maria João Ramalho, Joana Angélica Loureiro, Maria do Carmo Pereira

**Affiliations:** LEPABE, Department of Chemical Engineering, Faculty of Engineering of the University of Porto, 4200-465 Porto, Portugal; stephanie@fe.up.pt (S.A.); mjramalho@fe.up.pt (M.J.R.)

**Keywords:** neurodegenerative disease, bioactive compound, natural extract, β-amyloid peptide, tau protein, clinical trial, human studies, animal studies, in vitro studies

## Abstract

Alzheimer’s Disease (AD) is a neurodegenerative disorder related with the increase of age and it is the main cause of dementia in the world. AD affects cognitive functions, such as memory, with an intensity that leads to several functional losses. The continuous increase of AD incidence demands for an urgent development of effective therapeutic strategies. Despite the extensive research on this disease, only a few drugs able to delay the progression of the disease are currently available. In the last years, several compounds with pharmacological activities isolated from plants, animals and microorganisms, revealed to have beneficial effects for the treatment of AD, targeting different pathological mechanisms. Thus, a wide range of natural compounds may play a relevant role in the prevention of AD and have proven to be efficient in different preclinical and clinical studies. This work aims to review the natural compounds that until this date were described as having significant benefits for this neurological disease, focusing on studies that present clinical trials.

## 1. Introduction

Neurodegenerative diseases induce alterations in the central nervous system with psychological and physiological negative effects [1]. Alzheimer’s disease (AD) is known as a neurodegenerative disorder with major importance and the principal cause of dementia among the elderly [2,3]. Microscopically, intraneuronal neurofibrillary tangles (NFTs) and extracellular senile plaques (or amyloid plaques) characterize the AD. While senile plaques are constituted by extracellular deposits of β-amyloid (Aβ) peptide, the hyperphosphorylation and abnormal deposition of tau protein compose the NFTs [4].

Aβ derives from the amyloid precursor protein (APP), proteolytic cleavage of amyloid precursor protein (APP), an integral membrane protein that possesses the general properties of a cell surface receptor [5], by the consecutive action of β- and γ-secretases (amyloidogenic pathway). However, this amyloidogenic pathway can be stopped by the competition of α-secretase with γ-secretase (non-amyloidogenic pathway) [6]. The amyloid cascade hypothesis (ACH) suggests that the imbalance between the Aβ generation and its clearance causes the dysfunction and consequently cell death. Aβ polymerizes in a variety of structurally different forms including oligomeric, protofibrillar, and fibrils, forming the senile plaques [7]. Several findings suggest that oligomers play an important role in the ACH [8]. Nowadays, it is proved that Aβ oligomers, including protofibrils and prefibrils, are more toxic than fibrils [9]. Tau protein is also related with the ACH. First, tau monomers aggregate and form oligomers that aggregate into a β-sheet conformation, forming NFTs [10]. NFTs accumulate inside the neurons, resulting in their death. The ACH suggests that toxic concentrations of Aβ cause changes in tau protein and subsequent formation of NFTs, leading to synaptic and neuronal loss [11]. Though a direct relationship between the degree of AD and the amount of Aβ aggregates and tau levels have been established, numerous other mechanisms of neurodegeneration have been suggested, such as neuroinflammation [12], oxidative stress [13], genetic [14] and environmental factors [15]. So, there is an urgent need to develop efficient therapies that target the various pathogenic mechanisms associated with AD. Based on these mechanisms, different therapeutic molecules can act through different pathways [16,17,18]. However, the currently available medications only control the symptoms in an early stage of the disease [11].

**Figure 1:**
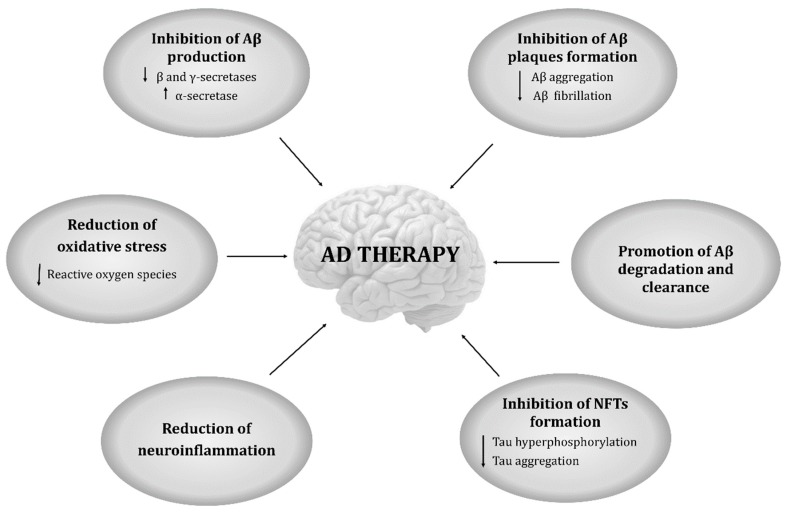
Schematic representation of the several mechanisms associated with Alzheimer’s Disease (AD) therapy. Down and up oriented arrows indicate the decrease and the increase of the fenomena, respectively.

Therefore, it is fundamental to seek for new strategies for AD therapy [19,20,21,22]. Natural compounds were the first molecules used as therapeutic agents [23]. Nowadays, the study of these natural compounds revealed that they present neuroprotective effects, arousing an increasing interest in the scientific community and in the pharmaceutical industry [24,25]. A diversity of natural compounds from different origins was described to be suitable to prevent and attenuate several pathologies, including neurological diseases, such as AD [26,27,28]. Several in vitro and in vivo studies have proven the therapeutic potential of natural compounds, however, just a small percentage has reached the clinical trials stage [29]. Since several causes are related with this disease, the preventive properties of the natural compounds can be associated with several mechanisms as shown in Figure 1 [6,30,31,32,33,34].

In this review, the natural compounds already in clinical trials phase are described and the reported results are presented and discussed. Other natural compounds with known potentially beneficial effects in AD in a preclinical development stage with in vitro and in vivo studies are also described. For preclinical studies, only the most recent reported works are cited. The systematic literature search was conducted using PubMed, Science direct, Google Scholar, Scopus and Web of Science as online databases until April 2019. Only papers written in English were considered with unlimited publication date.

## 2. Natural Compounds in Clinical Trials and Their Effects on AD

Natural compounds are an emerging approach for AD therapy. For the assessment of their therapeutic efficiency and potential side effects, human trials have been performed in the last years. The first natural product studied in a clinical trial was nicotine in 1992. However, no clinical trials were performed in the last two decades for this molecule. During the 90s, several other compounds were studied in clinical trials for AD therapy, such as vitamins. These molecules are still being tested in human trials up until this date. In the last years, other natural compounds are gaining interest by the scientific community and have achieved the clinical trials phase, such as bryostatin, which effects started to be evaluated in humans in 2017. A detailed report of these findings is described below. The natural compounds were divided into two groups: bioactive compounds and natural extracts, and they are summarized in Table 1 and Table 2, respectively. Here, a bioactive compound refers to a therapeutic molecule while a natural extract is the mixture of several molecules. The compounds are listed from the ones with more participants and longer duration.

### 2.1. Bioactive Compounds

**Figure 2:**
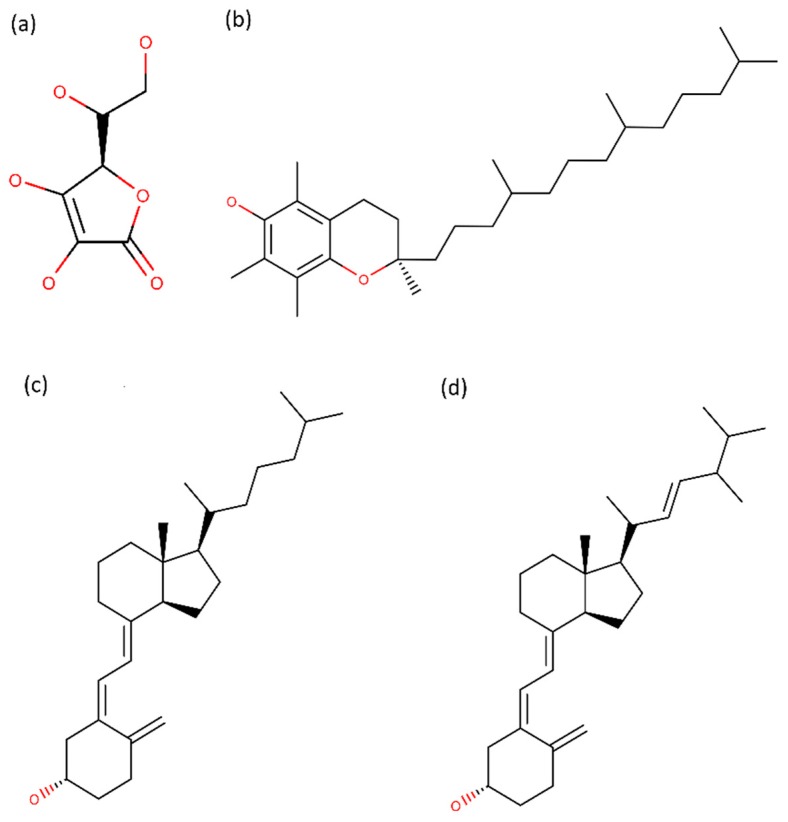
Chemical structures of: (**a**) vitamin C, (**b**) vitamin E, (**c**) vitamin D_3_ and (**d**) vitamin D_2_.

Vitamins have been described as therapeutic compounds for AD. Among them, vitamin C, E and D have aroused great interest. Vitamin C (Figure 2A) is found in several vegetables and fruits, mostly citrus fruits. In vivo studies reported that vitamin C prevented the neuroinflammation [81] and the brain oxidative damage due to its potent antioxidant activity [82]. Also, it was observed in an AD mouse model that Vitamin C reduced the Aβ oligomers formation and tau phosphorylation, improving the behavioral decline. The reduction of Aβ levels [83] and Aβ plaque burden [84] was also observed in vivo. 

On the other hand, vitamin E, which is present in several fruits and vegetables (Figure 2B), also showed in vivo antioxidant and anti-inflammatory effects [85]. Other in vivo study revealed that vitamin E reduced the Aβ levels [86].

Other vitamin with reported beneficial effects for AD, is vitamin D. Adding to several benefits of vitamin D [87], its therapeutic effect in AD has also been studied in last years. Although the major source of vitamin D is sunlight exposure (vitamin D_3_, Figure 2C) [88], around 20% can be obtained from food, including fatty fish and fish-liver oils (vitamin D_2_, Figure 2D) [89]. In vivo studies revealed that vitamin D is an anti-inflammatory compound [90] with the ability to inhibit the activity of β and γ-secretases, reducing the Aβ production and amyloid plaques and to increase the Aβ degradation [91]. As result, an improvement on learning and memory performance was verified in AD rats [92,93]. Also, low plasma Aβ is linked to the incidence of AD. 

Clinical trials revealed that vitamin D increased plasma Aβ in mild cognitive impairment patients, suggesting a reduction in Aβ levels in the brain. In fact, Miller et al. (2016) studied the effect of vitamin D supplementation on the plasma levels of Aβ in eight patients over 60 years old in a pilot study. Patients were randomly divided in two groups, treatment and placebo groups. Patients from the treatment group were administered with 50,000 IU per week for eight weeks. The obtained results showed that vitamin D intake increased plasma Aβ levels, suggesting a decrease in Aβ brain levels [35].

SanMartin et al. (2017) evaluated the role of vitamin D in the Aβ clearance from the brain. Patients with mild cognitive impairment and very early AD (*n* = 47) were orally supplemented with vitamin D at 50,000 IU once a week for six weeks, followed by 1500–2000 IU daily for 18 months. The obtained results showed that lymphocyte susceptibility to death, Aβ plasma levels and cognitive status improved after six months of vitamin D supplementation in cognitive impairment patients, but not in very early AD patients. Thus, supplementation with vitamin D proved to be beneficial in cognitive impairment patients. The lack of effects in very early AD patients suggest that vitamin D intake is not able to delay the progression of the disease in a more advanced stage [36].

Co-therapy with vitamin D and other molecules for AD therapy has also been explored in clinical trials. In fact, Annweiler et al. (2012) conducted a double-blind, placebo-controlled pilot trial with 43 white patients over 60 years with moderate AD symptoms [37]. The main goal of this trial was to evaluate the combination of neuroprotective effects of memantine and vitamin D in preventing neuronal loss and cognitive decline. Memantine was selected because is one of the most prescribed drugs for AD therapy [94]. Patients were randomly divided in three groups, being administered with memantine plus vitamin D (*n* = 8), or memantine alone (*n* = 18), or vitamin D alone (*n* = 17). Patients were administered with drugs for 24 weeks. Memantine was administered orally at 5 mg per week for the first four weeks and then 20 mg per day for the rest of the trial. Patients received a drinking solution of vitamin D at 100,000 IU every four weeks. After the study, patients co-treated with memantine and vitamin D showed better cognitive performance than patients treated with vitamin D or memantine alone [37].

Co-supplementation with vitamin D and other natural compounds was also studied in clinical trials. In fact, Galasko et al. (2012) conducted a double-blind, placebo-controlled clinical trial to evaluate what antioxidant supplementation affected the levels of AD’s histopathological marks, such as Aβ peptide and tau protein [38]. Patients with mild to moderate AD (*n* = 78) received placebo or daily supplement containing 800 IU of vitamin E, 500 mg of vitamin D, 900 mg of α-lipoic acid and 400 mg of coenzyme Q for 16 weeks. The attained results showed that the co-supplementation did not affect amyloid or tau levels, but a reduction on levels of an oxidative stress biomarker, the cerebrospinal fluid F2-isoprostane, was verified.

Also, co-supplementation with multivitamins was evaluated in clinical trials. In fact, Kontush et al. (2001) evaluated the efficiency of supplementation with both vitamin E and vitamin C to decrease oxidation of lipoproteins in AD patients [39]. Lipid oxidation is related with AD progression. Twenty patients with AD were randomly divided in two groups. The first group received a daily supplement for one month of 400 IU vitamin E alone, and the second group received a daily combination of 400 IU vitamin E and 1000 mg of vitamin C. The obtained results proved that combined supplementation was more efficient in maintaining active doses of vitamins in the plasma and decreasing lipid oxidation.

Co-therapy of different drugs with vitamin E was also studied in clinical trials. Sano et al. (1997) evaluated the effects of vitamin E and selegiline co-administration [40]. Selegiline is a monoamine oxidase inhibitor, that prevents dopamine degradation [95]. For that, a double-blind, placebo-controlled clinical trial was conducted with 341 patients with moderate AD’s symptoms for two years. The patients were randomly divided in four groups, a placebo group, one receiving vitamin E, one receiving selegiline, and another one receiving both drugs. Vitamin E was daily administered at a dose of 2000 IU per day, and 10 mg of selegiline daily. Co-therapy proved to efficiently slow the progression of the disease [40].

The combined effect of donepezil and vitamin E was also studied. Donepezil is a drug used for AD therapy to control the symptoms. To compare the effects of this drug with vitamin E on the outcome effects on patients with mild cognitive impairment, a double-blind, placebo-controlled clinical trial was conducted by Petersen et al. (2005) [41]. Patients over the age of 55 (*n* = 769) were randomly divided in three groups, placebo, vitamin E alone or donepezil alone. The daily dose of vitamin E was 1000 IU, and after six weeks the dose was increased to 2000 IU, for five years. Vitamin E proved to not be able to delay the disease progression. 

Dysken et al. (2014) studied the combination effects of vitamin E and memantine [42]. For that, a double-blind, placebo-controlled clinical trial was conducted with 613 patients with mild to moderate AD’s symptoms for five years. The patients were randomly divided in three groups, one receiving vitamin E, one receiving memantine, and another one receiving both vitamin E and memantine. The used doses for vitamin E were 2000 IU per day, and 20 mg of memantine daily. Treatment with vitamin E alone proved to be more efficient in slowing disease cognitive decline comparatively with the placebo group. However, no differences were verified for co-therapy comparatively with treatment with memantine alone. 

Kryscio et al. (2017) intended to assess if vitamin E and selenium intake could prevent dementia in healthy men over 60 [43]. Although no evidence exists to support the use of selenium in the treatment of AD, some works suggest that this product has a preventive potential [96]. A double-blind, placebo-controlled clinical trial involving 3786 male patients was conducted for 13 years. The participants were randomly divided into four groups. The first group received vitamin E, to the second only selenium was administered, the third group received a combination of vitamin E and selenium, and the fourth received placebo. The conclusions of this trials were that neither of the supplementation regimen proved to be able in preventing dementia [43].

**Figure 3:**
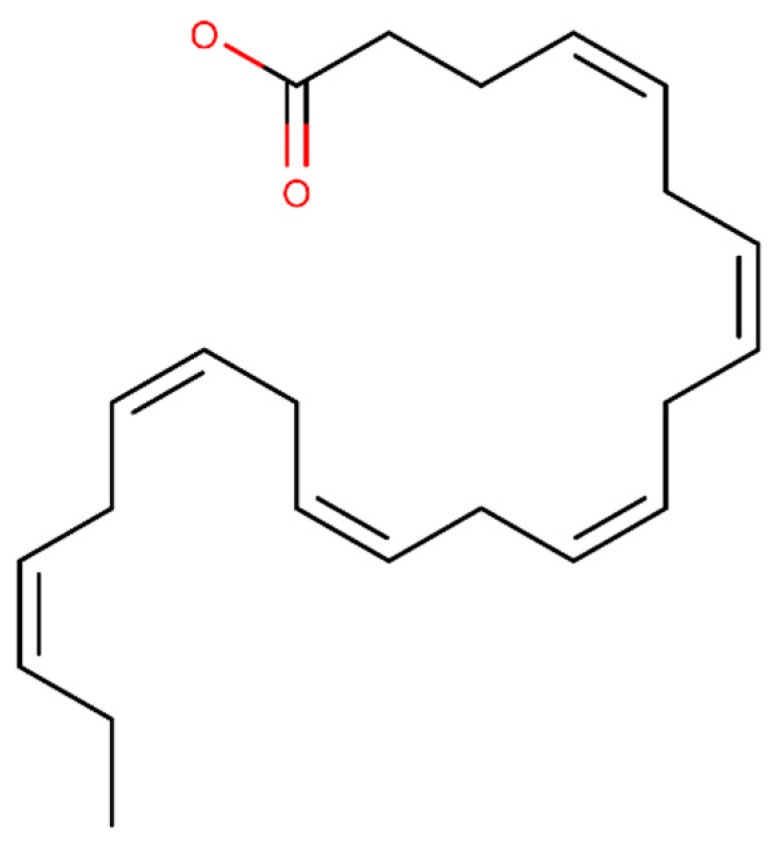
Chemical structure of docosahexaenoic acid (DHA).

Docosahexaenoic acid (DHA) is a polyunsaturated fatty acid from marine fish and algae [97] and its structural formula is presented in Figure 3. DHA demonstrated to have an antioxidant activity reducing the lipid peroxide and reactive oxygen species (ROS) levels in the brain of AD rats, improving the learning [98]. In addition, in vivo experiments showed that DHA reduces the Aβ levels, Aβ accumulation and plaque burden [99]. Some in vitro experiments demonstrated that DHA decreases the β- and γ-secretase activity and increases the α-secretase activity [100]. An in vitro study suggests that DHA reduced soluble Aβ oligomers levels and inhibited the formation and polymerization of Aβ fibrils [101]. Furthermore, DHA stimulated the Aβ degradation [102] and disaggregation of preformed Aβ fibrils in vitro [103].

The effects of supplementation with DHA in AD patients were studied in different clinical trials. In fact, Freund-Levi et al. (2006) conducted a double-blind, placebo-controlled clinical trial with 204 AD patients [44]. The main goal of this study was to evaluate the efficacy of dietary co-supplementation of DHA with other fatty acid, the eicosapentaenoic acid, on the cognitive functions of patients with mild to moderate AD. The patients were randomly divided in two groups, treatment and placebo. Patients on treatment group received a daily dose of 1.7 g of DHA and 0.6 g of eicosapentaenoic acid for six months. After this period, all patients received fatty acid co-supplementation for six more months. Despite the treatment being safe and well tolerated, the supplementation with these fatty acids did not delay the rate of cognitive decline of the patients.

Quinn et al. (2010) conducted a double-blind, placebo-controlled clinical trial to evaluate the efficacy of supplementation with DHA on the cognitive and functional decline in AD patients [45]. A daily dose of 2 g of DHA or placebo was administered to 295 patients for 18 months. The extent of brain atrophy was measured, and the results showed that DHA did not alter the patients’ condition. The attained results also proved that administration of DHA did not slow the rate of cognitive and functional decline. 

The same group conducted a double-blind, placebo-controlled, clinical study in the same year to evaluate the ability of DHA to improve the cognitive functions of 485 participants with age-related cognitive decline [46]. The subjects were randomly assigned to a daily oral administration of 900 mg of DHA orally or placebo for 24 weeks. The attained results proved that supplementation with DHA improved cognitive health, since the participants showed enhanced learning and memory functions.

Lee et al. (2013) studied the effects of DHA administration using fish oil on the cognitive function in patients over 60 diagnosed with mild cognitive impairment [47]. The participants (*n* = 36) were randomly divided in two groups, placebo and treatment group. The treatment group was orally administered with 430 g of DHA three times a day, for one year. No significant side effects were verified, suggesting the potential of DHA to improve memory functions. However, studies with more patients and longer intervention periods, are necessary to define the optimal dosage.

**Figure 4:**
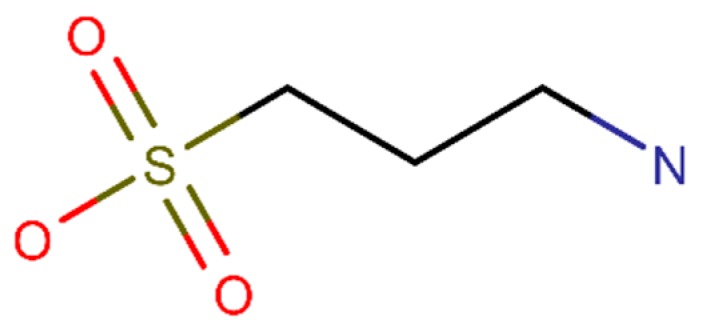
Chemical structure of homotaurine.

Homotaurine, also known as tramiprosate, is an aminosulfonate metabolite extracted from marine red alga *Grateloupia livida* and its structural formula is presented in Figure 4 [104]. In in vitro experiments, homotaurine proved to efficiently inhibit the Aβ aggregation [105] and reduce the Aβ plaque formation. This compound was also able to reduce the Aβ levels in vivo [106]. Additionally, the compound stabilized Aβ monomers and inhibited the Aβ oligomers formation in vitro [107]. 

Aisen et al. (2011) conducted a phase III double-blind, placebo-controlled trial with 1052 patients with mild to moderate AD symptoms to evaluate the effect of homotaurine in slowing AD progression [48,49]. This compound was the first inhibitor of Aβ aggregation that has reached a phase III clinical trial. The participants were randomly divided in three groups. The first group was the placebo group, and the other two groups received daily treatment with homotaurine at dose of 100 and 150 mg for 78 weeks, respectively. The authors proved that homotaurine administration had beneficial effect on cognition [108,109] 

The safety and tolerability of this compound administered to 58 patients with mild to moderate AD symptoms, were studied previously in a phase II clinical trial conducted by the same group [50]. Patients received placebo, 100 or 150 mg of homotaurine for three months. No harmful effects on vital signs were verified and the most frequent side effects were nausea, vomiting, and diarrhoea. 

Martorana et al. (2014) conducted a study with 10 patients with mild cognitive impairment with ages between 59 and 74 [51]. The participants were administered daily with 100 mg of homotaurine for four weeks. The obtained results showed that homotaurine improved the central cholinergic transmission.

**Figure 5:**
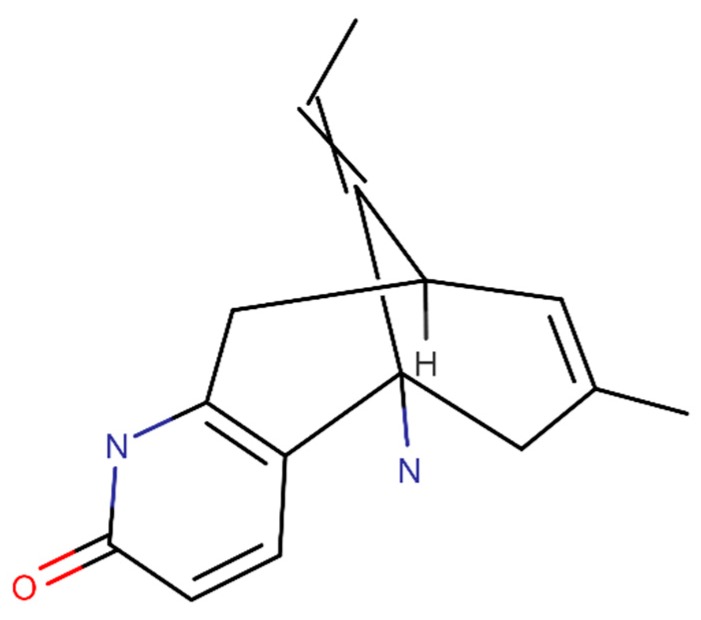
Chemical structure of huperzine A.

Huperzine A is isolated from *Huperzia serrata* (Thunb.) Trevis. (Lycopodiaceae) and its structural formula is presented in Figure 5. This compound demonstrated to have antioxidant properties. Huperzine A was able to reduce ROS and lipid peroxidation in an AD rat model [110]. Also, this product presents the in vitro ability to increase the α-secretase activity, significantly decreasing the Aβ levels, suggesting a blocking action in the Aβ production [111]. 

Xu et al. (1995) evaluated the efficacy and safety of huperzine A in AD patients. Four tablets of huperzine A (200 µg) or placebo were administered orally to 103 patients, twice a day, for eight weeks [52]. The results showed that the administration of huperzine A improved the memory and behaviour of AD patients. Also, the obtained results for the compound were better than for placebo. Huperzine A did not induce side effects. 

To further compare the efficacy and safety of huperzine A administered into capsules and tablets in AD patients, the same group conducted a new trial four years later [53]. In this study, 200 µg of huperzine A or placebo into capsules and tablets were administered twice a day to 60 patients, for 60 days. Both groups revealed a reduction in ROS levels in the plasma and erythrocytes of AD patients, without side effects besides nausea. This trial suggests that huperzine A in capsules and tablets is safe to be used in AD patients. 

Later, Rafii et al. (2011) studied the safety and efficacy of two concentrations of huperzine A, 200 and 400 µg twice a day, in patients with mild to moderate AD in a phase II clinical trial [54]. Placebo or huperzine A was administered to 177 patients for 16 weeks. The results demonstrated that at 400 µg/day huperzine A was not efficient, not being able to treat AD. However, at the concentration of 800 µg/day, the compound improved the cognition of AD patients. Huperzine A was safe at both studied doses. 

**Figure 6:**
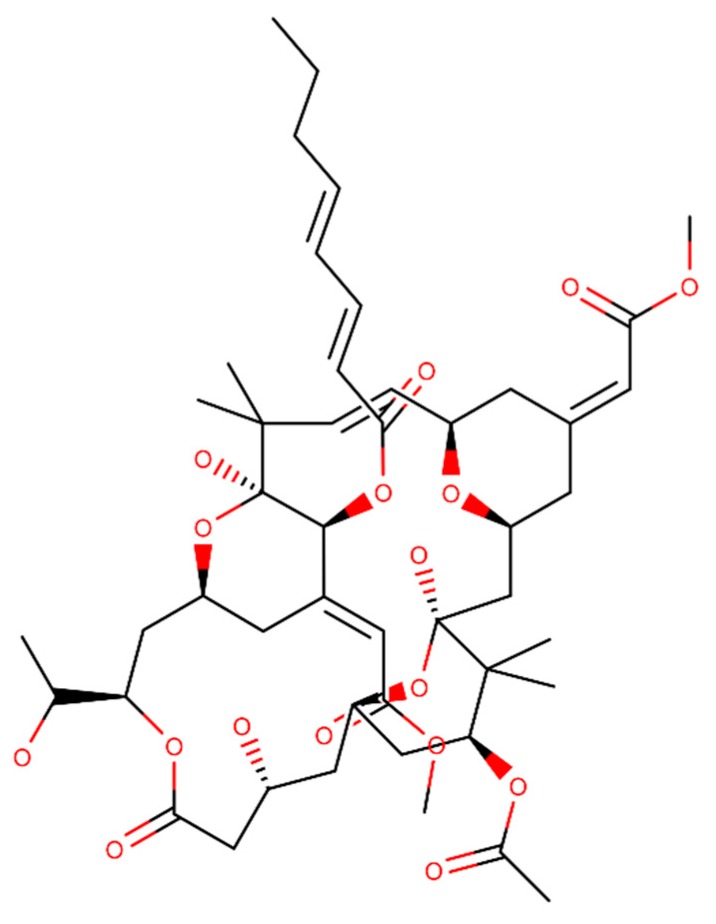
Chemical structure of bryostatin.

Bryostatin is a macrolide lactone extracted from *bryozoan Bugula neritina* [112]. The structural formula of the compound is presented in Figure 6. An in vivo study showed that bryostatin reduced the Aβ production by the stimulation of α-secretase activity, reducing the mortality of AD mice model [113]. Also, bryostatin revealed to enhance the learning and memory in AD mice model [114].

Recently, Nelson et al. (2017) evaluated the safety, tolerability and effects on cognitive function of bryostatin on AD patients in a phase II clinical trial [55]. A single dose of bryostatin at 25 µg/m^2^ was administered to six patients, while three patients received placebo. Bryostatin proved to improve cognitive functions and to be safe and well tolerated. 

Another phase II clinical trial was performed with the same goals [56]. Farlow et al. (2018) administered 20 or 40 µg of bryostatin or placebo to 150 AD patients, for 12 weeks. This study confirmed the safety of both doses of bryostatin. Also, an improvement of cognitive functions was observed using doses of 20 µg of bryostatin. 

**Figure 7:**
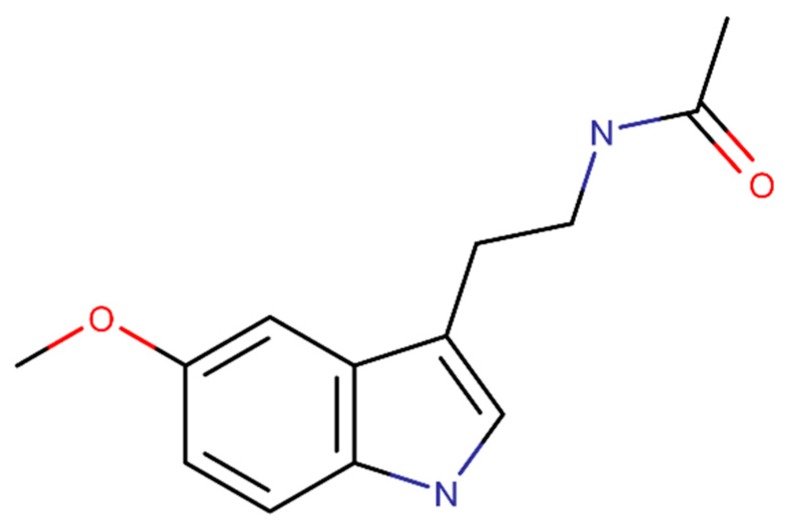
Chemical structure of melatonin.

Melatonin is collected from animals, plants, fungi and bacteria and its structural formula is presented in Figure 7. This compound demonstrated to have antioxidant properties due to its ability to decrease ROS in vivo [115]. In addition, an in vivo study reported the beneficial effects on neuroinflammation [116]. Further, an in vitro study proved the ability to inhibit the β-sheet conformation and, consequently, Aβ fibrils [117], decreasing the Aβ levels in the brain of AD rat model [118]. Another in vitro study proved that melatonin inhibits β- and γ-secretase activity and enhances the α-secretase activity, blocking the Aβ monomers production [119]. 

Brusco et al. (1998) evaluated the efficacy of melatonin in monozygotic twins with AD, with similar cognitive and neuropsychologic impairments [57]. Only one of the twins orally received daily 6 mg of melatonin for 36 months. The results suggest that melatonin improved the memory of the treated patient. Also, the clinical evaluation revealed that the twin that did not receive the treatment presented a more advanced state of the disease. 

Later, the same group studied the effect of melatonin in cognitive dysfunctions of 14 AD patients [58]. The patients received 9 mg of melatonin daily for 22 to 35 months. The results showed an improvement in cognitive functions, after the treatment. 

The same results were obtained by Furio et al. (2007) that performed a clinical trial with 50 outpatients diagnosed with mild cognitive impairment, where half of patients received 3 to 9 mg of melatonin for 9 to 18 months [59]. 

Wade et al. (2014) investigated the ability of 2 mg of melatonin to improve the cognitive functions of patients with mild to moderate AD [60]. Melatonin or placebo was administered to 80 patients for 24 weeks. Placebo was also administered two weeks before and after melatonin treatment. The results revealed an improvement in cognitive functions of AD patients treated with melatonin, comparing to placebo. Also, treatment was safe for both groups. Thus, these clinical trials suggested that melatonin administration can be a suitable therapeutic strategy for the treatment of AD.

**Figure 8:**
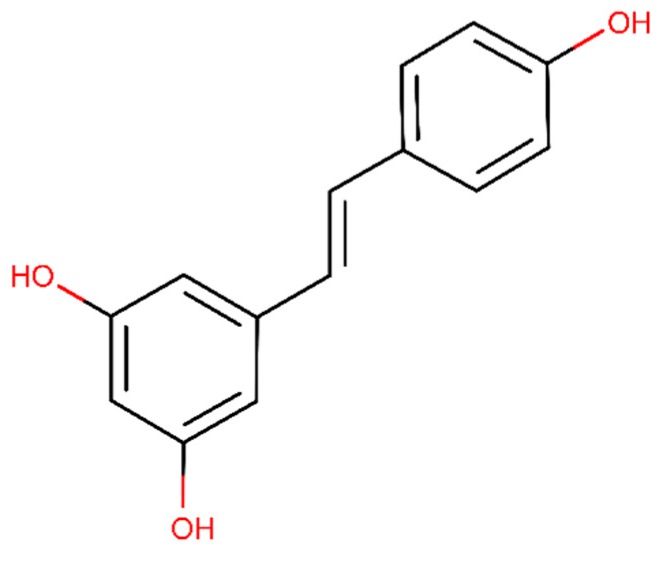
Chemical structure of resveratrol.

Resveratrol is a naturally occurring non-flavonoid polyphenol present in grapes (*Vitis vinifera* L. (Vitaceae)) and red wine and its structural formula is presented in Figure 8 [120]. In vitro experiments demonstrated that resveratrol induces the inhibition of studies proved a reduction of Aβ fibrils formation [121] and induced the in vitro Aβ disaggregation by an intracellular proteasomal action [108]. In vitro results showed that resveratrol has the ability to reshape toxic aggregates into a non-toxic aggregate type [109]. As result, resveratrol decreased the Aβ levels [122] and plaque levels in brain of AD rats [123]. In addition, in vivo evidence suggests that resveratrol has anti-inflammatory [122] and antioxidant effects [124]. Also, an in vitro study showed that resveratrol prevents the tau hyperphosphorylation [125].

Turner et al. (2015) performed a phase 2 clinical trial for 52 weeks in mild to moderate AD patients. The group studied the safety, tolerability and the ability of resveratrol to reduce the biomarkers of the disease (Aβ and tau). Here, 119 individuals were orally administered once a day with placebo or 500 mg of resveratrol, with an increase of 500 mg each 13 weeks. Although this study suggests that resveratrol can cross the blood-brain barrier (BBB), the results were not satisfactory. Besides inducing some side effects like nausea, diarrhea, and weight loss, the brain volume and biomarkers levels were lower in the placebo group than resveratrol group [61].

Recently, Zhu et al. (2018) evaluated the safety, tolerability and efficacy of a mixture containing 5 mg of resveratrol, 5 g dextrose and 5 g of malate. Fifteen mL of the mixture or placebo were orally administered twice a day to 39 patients with mild to moderate AD for one year. The administration was done together with an 8 oz glass of commercial grape juice. The results revealed that the preparation was safe and well tolerated. However, no evidence was observed concerning the efficacy of the product for AD therapy [62].

**Figure 9:**
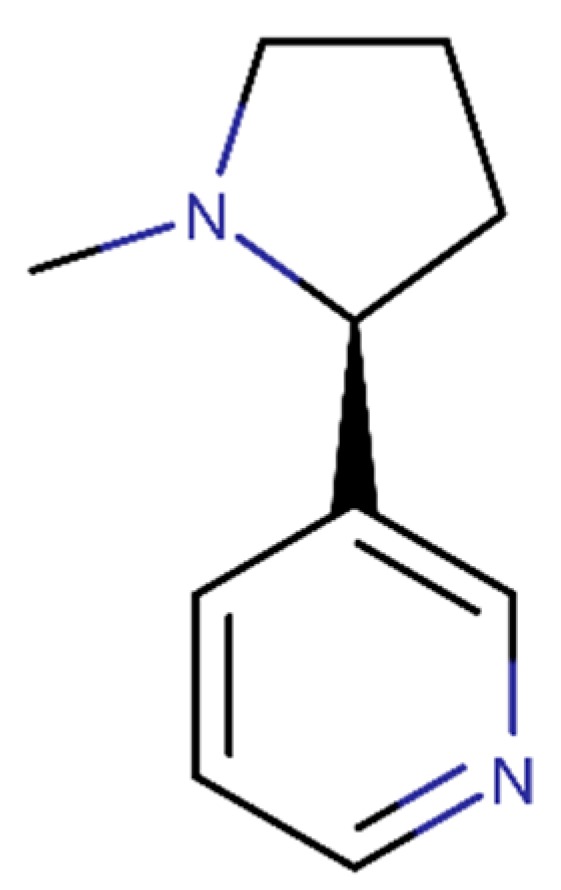
Chemical structure of nicotine.

Nicotine is extracted from the tobacco plant leaves (*Nicotiana tabacum* L., Solanaceae) and its structural formula is presented Figure 9. Nicotine presents the ability to delay the amyloidogenesis by inhibiting the β-sheet structures in vitro [126], decreasing in vivo β-secretase expression [127] and inhibiting in vivo Aβ aggregation [128]. An in vitro study revealed that nicotine inhibits the Aβ fibrils formation and their length, and disaggregate Aβ fibrils [129], causing an in vivo decrease of Aβ [127] and plaque amounts [128]. In addition, an in vitro study suggested valuable effects of nicotine due to their antioxidant properties [130]. Also, the decrease of APP containing Aβ peptide observed in in vivo experiments can be the reason to the diminution of Aβ and amyloid plaque levels [131]. 

Jones et al. (1992) studied the effect of nicotine on AD patients [63]. Three acute doses of nicotine (0.4, 0.6 and 0.8 mg) were subcutaneously administered to 22 AD patients and 48 controls. The results revealed that nicotine improved the perceptual and visual attentional deficits observed in AD patients. 

The effect of nicotine on behaviour, cognition, and physiology of six AD patients was evaluated in a pilot study proposed by Wilson et al. (1995) [64]. Placebo, nicotine and washout were sequentially administered for seven, eight and seven days, respectively. After nicotine administration, an improvement in learning was observed, which persisted with washout. Memory, behaviour and cognition were not affected. Also, the safety of nicotine was proved.

The clinical and neuropsychological effects of nicotine was evaluated in eight AD patients by White et al. (1999) [65]. Transdermal nicotine was administered for two periods of four weeks, separated by two weeks of washout. A nicotine patch was used daily for 16 h with the following doses: 5 mg/day in the first week, 10 mg/day in the second and third week, and finally, 5 mg/day in the fourth week. The results suggest that nicotine significantly improved the attentional performance. However, the limited sample of the study does not allow conclusive results.

**Figure 10:**
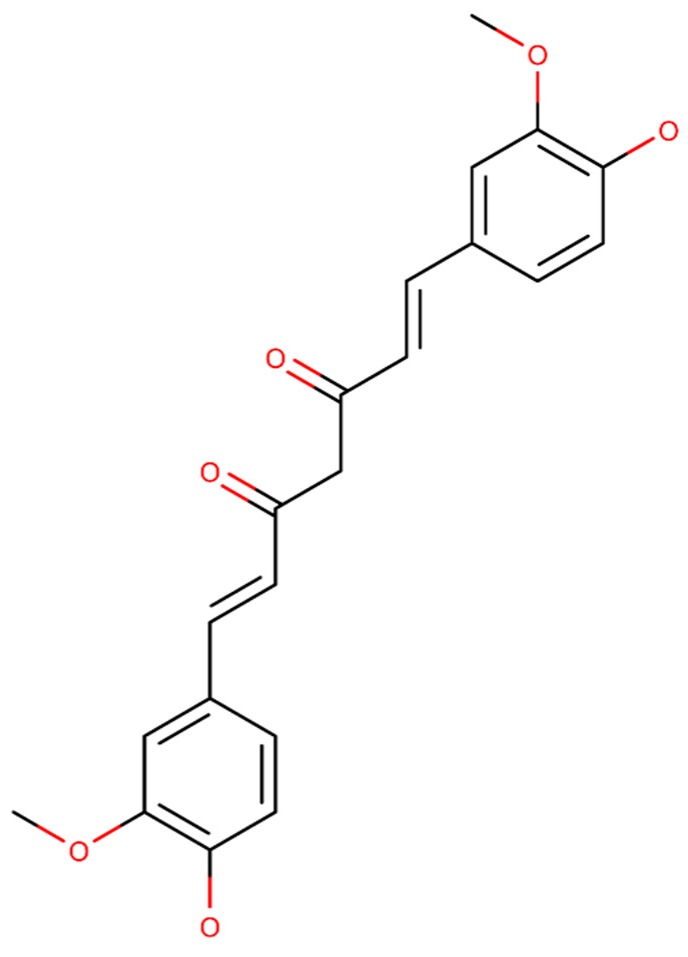
Chemical structure of curcumin.

Curcumin is an active component founded in the root of *Curcuma longa* L. (Zingiberaceae) and its structural formula is presented in Figure 10. This compound presents the in vivo ability to prevent the Aβ aggregation and disaggregate preformed Aβ fibrils [132,133]. Also, curcumin presents in vitro and in vivo anti-inflammatory and antioxidant beneficial effects, respectively [134,135]. Also, in vitro experiments showed that curcumin decreases β and γ-secretase levels [133,136,137]. As result, the spatial learning of AD rat model was improved, as well as the memory impairment [133]. 

Baum et al. (2008) performed a clinical trial to study the safety of curcumin on AD patients [66]. For six months, the authors administered 1 g, 4 g of curcumin or placebo in 34 AD patients. The results proved that curcumin did not produce side effects in AD patients, but the authors revealed the necessity of additional trials to confirm the efficacy of curcumin in AD treatment.

### 2.2. Natural Extracts and Other Natural Products

Ginkgo biloba (*Ginkgo biloba* L., Ginkgoaceae) has been studied as therapeutic drug for AD and other neurological diseases therapy. In vitro evidence revealed that ginkgo biloba extract can prevent Aβ aggregation, decrease Aβ fibrillogenesis and destabilize preformed fibril [138]. Substantial in vivo experimental evidence indicates that ginkgo biloba has antioxidant [139] and anti-inflammatory properties, ameliorating the cognitive and memory impairment in an AD rat model [140]. In vivo studies showed that ginkgo biloba favors the non-amyloidogenic via of APP by increasing α-secretase activity, inhibiting the Aβ production [141,142]. 

Several clinical trials have been carried out in the last 10 years to test the viability of the compound in treating patients with dementia. Bachinskaya et al. (2011) examined the effect of gingko biloba extract EGb 761^®^ on neuropsychiatric symptoms of dementia [67,68]. Outpatients with mild to moderate dementia (AD with or without cerebrovascular disease or vascular dementia) (*n* = 410) were considered in this study. Patients received 240 mg of extract or placebo once daily for 24 weeks. The treatment with gingko biloba was safe and improved the neuropsychiatric symptoms, which include apathy, irritability, depression, among others. 

Also, with the same conditions, Herrschaft et al. (2012) revealed that the treatment with gingko biloba improved the cognition and the life quality of patients [69]. 

Ihl et al. (2012) performed a similar 24-week randomised controlled trial involving 404 outpatients [70]. Patients were diagnosed with AD (*n* = 333) or vascular dementia (*n* = 71). In addition to confirming the improvement of neuropsychiatric symptoms observed in the previous trial, the extract improved the cognitive functions and functional abilities of patients. 

Gavrilova et al. (2014) also conducted a clinical trial to study the effects of gingko biloba in neuropsychiatric symptoms and cognition in 160 patients with mild cognitive impairment [71]. The patients received 240 mg of EGb 761^®^ or placebo for 24 weeks. The trial proved that the extract improved the neuropsychiatric symptoms and cognitive functions of patients. Also, the extract was safe and well tolerated. Taking together, the last clinical trials proved that a 240 mg daily dose of ginkgo biloba extract is safe in the treatment of dementia.

Saffron (*Crocus sativus* L., Iridaceae) is a stem-less herb with antioxidant [143] and anti-inflammatory activities in vivo [144]. This product inhibited the in vitro Aβ aggregation and fibrillogenesis [145]. 

Akhondzadeh et al. (2010) evaluated the efficacy of 30 mg saffron in the treatment of mild to moderate AD [72]. Saffron or placebo were orally administered daily for 16 weeks, to 46 patients. The phase II study showed that the administration of saffron improved the cognition and memory of AD patients. Also, no side effects differences were observed with saffron or placebo administration. Thus, saffron seems to be safe in the treatment.

Lemon balm (*Melissa officinalis* L., Lamiaceae) from the mint family that is native to Europe with antioxidant activity in vitro [146]. In vivo studies proved the ability of lemon balm extract to improve the memory of an AD model, probably due to the inhibition of β-secretase activity [147]. To assess the efficacy and safety of *Melissa officinalis* extract on patients with mild to moderate AD, Akhondzadeh et al. (2013) administered to 40 patients 60 drops of extract or placebo, for four months [73]. The results proved that *Melissa officinalis* extract ameliorated the cognition and agitation of AD patients. 

Green tea (*Camellia sinensis* (L.) Kuntze, Theaceae) from steaming and drying of leaves of the *Camellia sinensis* plant proved to be a rich source of antioxidants in in vivo studies [148]. In addition, the green tea prevented the spatial learning and memory destruction in an AD mice model by decreasing Aβ oligomers levels [149] and hyperphosphorylated tau protein [150]. 

Recently, Arab et al. (2016) developed a clinical trial with 30 patients to study the antioxidant activity of green tea in patients with severe AD and its ability to improve cognitive functions [74]. Patients received daily 2 g of green tea through the ingestion of pills, for two months. The results showed an improvement on cognitive functions, confirming the effects of the antioxidant activity of green tea. 

Papaya (*Carica papaya* L., Caricaceae) is a fruit often used in medicine that has amino acids, β-carotene, oligosaccharides and vitamins, with benefits in AD. 

A clinical trial performed by Barbagallo et al. (2015) studied the antioxidant activity of fermented papaya powder extract in AD patients [75]. AD patients (*n* = 20) received 4.5 g of extract daily for six months, while the 12 controls did not receive any treatment. The results showed that the supplementation with fermented papaya powder reduced the ROS generation and nitric oxide production in AD patients, with no significant changes in controls. Thus, the papaya can be used as antioxidant in the AD therapy.

Sage (*Salvia officinalis* L., Lamiaceae) is a medicinal plant with a long-standing reputation in European medical herbalism due to its anti-inflammatory and antioxidant properties observed in vivo [151]. 

Akhondzadeh et al. (2008) developed a clinical trial to evaluate the efficacy and safety of *Salvia officinalis* extract in the treatment of patients with mild to moderate AD [76]. Patients received daily 60 drops of sage extract or placebo for four months. The results showed that sage extract improved cognitive functions. Also, after the treatment, any group revealed side effects except agitation, that seems to be more pronounced in placebo group. This study proved that sage can be useful in the therapy of mild to moderate AD. 

Coconut (*Cocos nucifera* L., Arecaceae) demonstrated to be able to reduce the Aβ deposition and aggregation and the oxidative stress in a transgenic *Caenorhabditis elegans* AD model [152]. Coconut oil also enhanced the memory of rats [153]. Also, in vitro studies demonstrated that the coconut oil reduced de APP expression, decreasing the Aβ secretion [154] and protected neuronal cells against Aβ-induced neurotoxicity. 

Ortí et al. (2018) performed a clinical trial with 44 AD patients [77]. Half of individuals received daily 40 mL of coconut oil, distributed by the breakfast (20 mL) and lunch (20 mL), for 21 days. Before and after the oil administration, cognitive function was evaluated. The trial revealed that the patients treated with coconut oil demonstrated an improvement of cognitive functions. 

Apple (*Malus domestica* Borkh., Rosaceae) showed to be a promising approach to prevent AD. In vivo evidence demonstrated that the apple extract prevents the oxidative stress and reduces the Aβ levels, improving the memory of AD rats [155]. Besides, in vivo studies demonstrated that apple juice is able to reduce γ-secretase expression, which leads to the reduction of Aβ production [156]. 

Remington et al. (2010) performed an open-label pilot clinical trial with 21 patients with moderate to severe AD [78]. The authors administered two 4-oz of apple juice daily for one month. Although the results suggest that there was no modification in the degree of dementia, a significant improvement in behavioural and psychotic symptoms was observed, with reduction of anxiety, agitation, and delusion. This study suggests that the supplementation with apple juice can attenuate the AD-related decline.

Blueberry (*Vaccinium myrtillus* L., Ericaceae) is a fruit composed by several polyphenols named anthocyanins, with antioxidant [157] and anti-inflammatory properties in vivo [158]. In vitro works suggested that blueberries increase the Aβ clearance [159] and inhibit the Aβ aggregation, decreasing the amount of toxic species [157]. As a result, an improvement in cognitive functions and motor performance was observed in an AD mouse model [160]. 

Krikorian et al. (2010) evaluated the effects of daily administration of wild blueberry juice in a group of nine elderly subjects with early memory failures [79]. The daily consumption of blueberry juice was proportional with body weight, varying between 6 and 9 mL/kg. After 12 weeks of treatment, an improvement in learning was observed as well as a reduction of depressive symptoms. The study suggests that the blueberry supplementation can confer neuroprotection.

Colostrinin, a milk form produced by mammary glands [161], presents in vitro antioxidant and anti-inflammatory activities, and inhibits the Aβ fibrils formation and disassembles Aβ aggregates [162]. Also, the ability of colostrinin to inhibit tau phosphorylation and eliminate Aβ was proved in vitro [163]. 

The effect of colostrinin on AD patients was studied in a clinical trial conducted by Szaniszlo et al. (2009) [80]. Patients over 50 received 100 μg of colostrinin or placebo for 15 weeks. The results showed an enhancement in cognitive and daily function of AD patients treated with colostrinin. Thus, this compound can be a suitable approach for AD therapy.

## 3. Preclinical In Vivo Studies of Natural Compounds and Their Effects on AD

Besides the natural compounds that have been studied in clinical trials, several other products have proved to have a potential beneficial effect in AD therapy in a preclinical stage, namely in in vivo studies. The preclinical phase involving in vivo studies is conducted to assess if the new compounds are safe and effective, before they can proceed to the clinical trials phase. A detailed report of animal studies results is described below. The natural compounds were divided into two groups: bioactive compounds and natural extracts and organized by the number of mechanisms associated with AD therapy, from the highest to the least.

### 3.1. Bioactive Compounds

Epigallocatechin gallate (EGCG) is a polyphenol found in green tea with several neuroprotective effects in AD. In vivo evidence suggests that EGCG decreased β- and γ-secretase actions and enhanced the α-secretase activity, leading to the decrease of Aβ levels improving the memory [164]. Besides that, EGCG inhibited the in vitro Aβ aggregation [165] and the in vivo Aβ oligomerization [166]. Moreover, EGCG inhibited the in vitro tau aggregation [167] and increased the in vivo clearance of phosphorylated tau [168]. Lastly, EGCG has been reported in in vivo experiments to demonstrate antioxidant [169] and anti-inflammatory actions [170]. 

Retinoic acid is a terpenoid and a metabolite of vitamin A. In vitro studies revealed that retinoic acid inhibited Aβ fibrils formation and their extension and destabilized Aβ fibrils [171]. In vitro evidence demonstrated that retinoic acid decreases the Aβ levels by inhibiting β- [172] and γ-secretase [173] and increasing α-secretase activity [172]. An in vivo study reported the ability of retinoic acid reducing brain Aβ deposition, APP phosphorylation and tau phosphorylation. This work also proved the anti-inflammatory activity of this compound, improving the learning and memory of AD mice model [174].

Caffeine is perhaps the most consumed psychoactive compound. It is present in the coffee bean, but it can be also found in some teas, cocoa drinks, candy bars, among other herbs. In vivo studies suggest that caffeine reduced the β-secretase and γ-secretase levels, decreasing the Aβ production [175]. An in vitro study showed that the inhibition of the β-sheets conformation can be related with the ability of caffeine to reduce Aβ levels [176]. Also, it was observed in vivo that this natural product promotes Aβ clearance [177]. In vivo evidence suggested that caffeine have anti-inflammatory and antioxidant properties [178]. In vivo studies demonstrated that the improvement observed in the memory could result from hippocampal tau phosphorylation reduction [179]. 

Baicalein is a naturally occurring flavonoid from the roots of *Scutellaria baicalensis* Georgi (Lamiaceae). In vitro studies suggested that baicalein inhibits the ROS production, reducing the oxidative stress [180]. In vitro results proved that baicalein inhibits Aβ fibrillation and oligomerisation and disaggregates Aβ fibrils [181]. In vivo studies proved that baicalein is able to increase the α-secretase and decrease the β-secretase activities, reducing the Aβ production [182,183]. Also, the tau phosphorylation in AD model mice was prevented and the cognitive function improved [183]. 

Berberine is an isoquinoline alkaloid found in rhizoma coptidis, an herb frequently used in Chinese herbal medicine. In vivo evidence suggests that berberine inhibited the β-secretase expression, reducing the Aβ production. Also, berberine stimulated the Aβ clearance and inhibited the Aβ plaque deposition and hyperphosphorylation of APP and tau [184]. Berberine has been also described as having in vivo anti-inflammatory and antioxidative activities [185].

Kaempferol is a polyphenolic flavonoid found in fruits, vegetables and herbs. In vivo studies proved its antioxidant effect, improving the learning and memory of a transgenic drosophila AD model [186]. Also, in vitro evidence showed that kaempferol has anti-inflammatory activity [187], inhibits Aβ aggregation [188] and destabilizes Aβ fibrils [189]. Also, another in vitro study proved that kaempferol inhibits the β-secretase activity [190]. 

Quercetin is a flavonol, naturally occurring polyphenolic compounds present in fruits, vegetables and herbs. In vivo studies showed that quercetin improved the memory and cognitive impairments of an AD model and reduced the oxidative stress [191]. Moreover, in vitro evidence suggested that quercetin prevents the Aβ aggregation [192], inhibits the Aβ fibrils formation and destabilizes Aβ fibrils [193], decreasing the Aβ levels in brain of AD model mice [194]. Additionally, this compound was reported in in vivo studies as inhibitor of β-secretase and taupathy [195].

Fisetin is a flavonoid extracted from *Rhus succedanea* L. (Anacardiaceae) and also found in some fruits and vegetables. Fisetin proved to inhibit Aβ aggregation in vivo [196] and fibril formation in vitro [188], reducing the in vivo Aβ accumulation [197]. Also, an in vivo experiment described fisetin as a β-secretase inhibitor and anti-inflammatory product [197]. Additionally, fisetin promotes the in vitro degradation of phosphorylated tau [198] and reduced the in vivo tau hyperphosphorylation [197].

Oleuropein is a polyphenol present in extra virgin olive oil with antioxidant [199] and anti-inflammatory properties in vivo [200]. The Aβ levels and amyloid plaque load were reduced in vivo, resulting in an amelioration of cognitive functions [201]. Also, the compound inhibited the Aβ aggregation in vivo [200], favouring the formation of non-toxic aggregates in vitro [202]. Additionally, in vitro evidence suggested that oleuropein decreased the Aβ oligomers levels through the promotion of α-secretase activity [203]. Lastly, oleuropein was described as tau aggregation inhibitor in vitro [204].

Tannic acid is a polyphenol found in herbs and fruits. An in vivo experiment showed that tannic acid is a natural inhibitor of β-secretase with anti-inflammatory properties, preventing the cognitive impairment of AD mice [205]. One in vitro study affirmed that tannic acid inhibits Aβ formation associated with less amyloidogenic APP proteolysis, inhibits Aβ fibrils formation as their extension and still destabilizes Aβ fibrils [206]. Another in vitro study demonstrated that tannic acid inhibits the tau aggregation [207]. 

Crocin is a carotenoid mainly found in the stigma of saffron flower. In vitro experiments showed that crocin inhibits the Aβ fibril formation [208] through the inhibition of the Aβ fibrillogenesis [145]. Also, in vitro evidence suggests that crocin reduces the number of fibrils as well as their length [208]. An in vitro study confirmed that crocin can also disrupt Aβ aggregates [209]. Also, the therapeutic effects of crocin can be linked to its antioxidant [210] and anti-inflammatory activities [211] observed in in vivo studies.

Epicatechin represents one of the antioxidants from the flavonoids family. High amounts of this compound can be found in cocoa beans, green tea and grapes. In vivo data showed that epicatechin has antioxidant [212] and anti-inflammatory activities [213]. Further, in vitro studies suggest that epicatechin is an inhibitor of β-secretase [214]. As result, epicatechin decreased the Aβ levels in an AD mice model [212]. Also, epicatechin has the in vitro ability to inhibit tau aggregation [215] and fibril formation changing the secondary structure [216].

Gallic acid is a phenolic acid present in fruits, vegetables and herbs. Gallic acid proved to have antioxidant [217] and anti-inflammatory activities, improving the learning and memory in vivo [218]. Also, gallic acid can reduce the in vitro Aβ aggregation by the inhibition of conformational transition to β-sheet [219]. An in vivo experiment observed a reduction in Aβ levels after gallic acid administration due to the increase of α-secretase action, promoting the non-amyloidogenic route and consequently the decreases the Aβ oligomerization [220].

Ferulic acid is a phenolic compound naturally present in numerous fruits and vegetables. In vivo results revealed that ferulic acid is an antioxidant [221] and anti-inflammatory compound [222]. Also, it can reduce the in vivo Aβ production by reducing the β-secretase activity [222]. The decrease of β-sheets structures was also observed in an in vitro experiment, inhibiting the Aβ aggregation [223]. Additionally, ferulic acid decreased the Aβ deposition and improved the cognitive performance of an AD mouse model [224]. Also, ferulic acid decreased the Aβ fibrils levels in vitro [225].

Rutin is a bioflavonoid extracted from some vegetables and fruits. This product is a glycoside of the flavonoid quercetin with antioxidant and anti-inflammatory properties in vivo [226]. The same in vivo study showed that this compound inhibited the Aβ aggregation [226]. Also, rutin decreased the Aβ fibrils formation in vitro [193]. This can be due to its ability to inhibit the β-secretase activity in vitro [193]. Also, rutin disaggregated Aβ fibrils in vitro [193].

Salvianolic acid B is a phenylpropanol founded in the *Salvia miltiorrhiza* Bunge (Lamiaceae) root. In vivo experiments showed a strong antioxidant and anti-inflammatory activities, improving the memory and learning of an AD mouse model [227]. Also, salvianolic acid B inhibited the Aβ aggregation and disaggregated preformed Aβ fibrils in vitro [228]. Another in vitro work suggested that salvianolic acid B inhibits the β-secretase which leads to the inhibition of Aβ production [229].

Myricetin is a flavonoid extracted from several fruits, vegetables and herbs. In vitro proofs showed that myricetin prevents Aβ aggregation and consequent fibrillation [189,230] due to its capacity to inhibit β-secretase and increase the α-secretase activity [231]. Also, myricetin blocked the structural changes on Aβ in vitro, inducing a reduction in Aβ levels [231]. Also, the disaggregation of Aβ fibrils was observed in vitro [189]. As result, an in vivo study showed that myricetin enhanced the learning and memory impairments in an AD rat model [232].

Naringenin is a natural compound present in citrus fruits and tomatoes. It is the major flavanone constituent found in *Citrus junos* Siebold ex Tanaka, Rutaceae. An in vitro study revealed that naringenin inhibited the APP and β-secretase activity and reduced the levels of phosphorylated tau [233]. As result, brain levels of Aβ were reduced in vivo [234]. In vivo evidence also proved the antioxidant [235] and anti-inflammatory activities of the compound, improving motor coordination, learning and memory of AD rats [236].

Luteolin, a polyphenol flavonoid found in fruits, vegetables and herbs, exhibits potent anti-inflammatory activity in vitro [237] and antioxidant activity against induced-oxidative stress in a in vivo AD model [238], ameliorating the spatial learning and memory impairment [239]. An in vitro study also proved that this compound is a potent inhibitor of β-secretase [240]. Another in vitro study demonstrated that luteolin is able to reduce tau hyperphosphorylation [241]. 

Asiatic acid is a pentacyclic triterpene found in plants. Asiatic acid demonstrates an ability to inhibit the β-secretase and increase the α-secretase activity in vitro. Also, it demonstrates an ability to activate Aβ clearance [242], which explains the substantial reduction in Aβ levels in AD mice [243]. Numerous in vivo works suggest that asiatic acid has antioxidant properties, clearing free radicals and decreasing lipid peroxidation, improving the learning and memory [244].

Puerarin is an isoflavanone glycoside isolated from *Pueraria lobata* (Willd.) Ohwi (Leguminosae) used to treat some diseases. In vivo studies found that puerarin inhibited the tau phosphorylation and reduced Aβ levels, ameliorating the spatial learning and memory in an AD mice model [245]. The beneficial effects of puerarin were suggested in in vivo experiments to be connected to its ability to reduce the oxidative stress [246] and neuroinflammation [247]. 

Oleocanthal is one of the main active components of extra virgin olive oil. In vitro evidence suggests that this compound changes the structure of tau protein, inhibiting its aggregation [248] and fibrillization [249]. In vivo results proved that oleocanthal enhances the Aβ clearance, reducing the amyloid load. Also, the anti-inflammatory activity of the compound was verified [250].

Viniferin (trans ε-viniferin) is a polyphenol present in a variety of vines, including *Vitis vinifera* L., Vitaceae. In vitro evidence proved the anti-inflammatory [251] and antioxidant [252] activities of the compound. Also, viniferin disaggregated Aβ [251] and inhibited the Aβ aggregation, reducing the fibril formation [253].

Scyllo-inositol, also known as scyllo-cyclohexanehexol, is one of the stereoisomers of inositol, found in dogwood *Cornus florida* L. (Cornaceae) and coconut palm *Cocos nucifera* L. (Arecaceae). An in vivo study showed that this compound decreases the Aβ levels and inhibits the Aβ aggregation, improving the memory of AD rat model [254]. In vitro evidence demonstrated that scyllo-inositol induces structural modifications in Aβ, stabilizes Aβ oligomers and inhibits fibril formation [255].

Honokiol is a poly-phenolic product found in *Magnolia officinalis* Rehder & E.H.Wilson, Magnoliaceae. In vivo evidence suggested that honokiol is an antioxidant [256] and anti-inflammatory compound [257]. In vivo studies revealed that honokiol inhibits the β-secretase activity, reducing the Aβ production and senile plaque deposition. Also, the Aβ degradation was enhanced by honokiol [257]. As result, honokiol decreased Aβ-induced hippocampal neuronal apoptosis, improving learning and memory of AD mice model [256].

Apigenin is a flavonoid found in plants, fruits and vegetables. Numerous in vitro and in vivo works showed its anti-inflammatory [258] and antioxidant [259] properties, respectively. An in vivo experiment proved that apigenin changes APP processing by the β-secretase inhibition preventing the Aβ deposition and consequently, improving the memory impairments [259].

Caffeic acid is a phenolic acid present in food, beverages and Chinese herbal medicines with antioxidant and anti-inflammatory properties in vivo. This compound improved the learning of AD rat models [260]. In vitro studies showed that caffeic acid reduced the tau phosphorylation and protected the PC12 cells against Aβ-induced toxicity [261]. 

β-carotene belongs to the carotenoid family. One in vitro study reported that β-carotene has an anti-aggregation activity and destabilizes Aβ [171]. Another in vivo study demonstrated the β-carotene has the ability to reduce oxidative stress, by reducing the ROS production [262]. 

Rosmarinic acid is a phenolic carboxylic acid found in rosemary, lemon balm and peppermint, among others. An in vivo study proved that this compound has antioxidant properties, protecting an AD mouse model against memory deficits [263]. Also, rosmarinic acid inhibited the tau hyperphosphorylation [264] and fibrillization in vitro [265].

Nordihydroguaiaretic acid (NDGA) is a compound found in *Larrea divaricata* Cav. (Zygophyllaceae) with in vivo antioxidant properties [266]. An in vitro study reported that NDGA inhibits the Aβ fibrils formation, reducing the number of fibrils and small amorphous aggregates. Additionally, this compound disrupts Aβ fibrils [267].

Osthole is a coumarin isolated from *Cnidium monnieri* (L.) Cusson (Apiaceae). An in vivo study showed that this compound significantly enhanced the memory of an AD rat model, that can be linked to its antioxidant activity [268] and with a reduction of Aβ levels found in the brain. This reduction can be due to the inhibition of β-secretase in vitro [269]. Also, in vitro evidence suggests that this product decreases the phosphorylated tau levels [270].

Ellagic acid is a polyphenol extracted from *Punica granatum* L. (Lythraceae). An in vitro study proved that this compound inhibits of β-secretase activity preventing neurotoxicity [271]. Ellagic acid has antioxidant and anti-inflammatory properties, that improve learning and memory injuries in AD rat model [272].

Glycine betaine is an organic osmolyte, which could be isolated from vegetables and marine products. In vivo evidence revealed that glycine betaine reduces tau hyperphosphorylation and Aβ production, improving memory deficits [273]. Also, glycine betaine inhibited the β-secretase activity and activated the α-secretase activity in vitro, thereby inhibiting the Aβ production [274]. 

Hydroxytyrosol is a phenolic compound extracted from the olive leaf and oil. In vivo studies demonstrated that it is a compound with antioxidant and anti-inflammatory properties [275]. Also, hydroxytyrol showed to reduce the levels of Aβ plaques in an AD mice model [276].

l-theanine is an amino acid present in green tea. An in vivo work showed that l-theanine decreased the oxidative stress and the Aβ levels [277]. Also, this natural product proved to inhibit tau hyperphosphorylation in vitro [278].

13-Desmethyl spirolide C is a marine compound belonging to the cyclic imine group produced by the dinoflagellate *Alexandrium ostenfeldii* and accumulate in shellfish. An in vitro study revealed that 13-desmethyl spirolide C is a spirolide that can reduce intracellular Aβ accumulation and hyperphosphorylated tau levels [279]. The reduction of intracellular Aβ levels was also observed in an in vivo study [280].

Gossypin is a flavonoid found in *Hibiscus vitifolius* L. (Malvaceae) and has been reported in in vivo experiments to exhibit anti-inflammatory [281] and antioxidant actions [282].

Gypenosides are triterpenoid saponins extracted from *Gynostemma pentaphyllum* (Thunb.) Makino (Cucurbitaceae) and they are reported in an in vivo study to be products with antioxidant and anti-inflammatory activities, improving the cognitive impairment [283].

1,2,3,4,6-Penta-*O*-galloyl-β-d-glucopyranose (PGG) is a polyphenol and the main constituent of the *Paeonia x suffruticosa Andrews* (Paeoniaceae) root, a tree peony native to China and used in traditional medicine practices. In vivo experiments proved that PGG inhibits the Aβ oligomerization, which prevents Aβ fibril formation, resulting in the decrease of Aβ levels and improvement of memory. PGG is also able to promote the destabilization of Aβ fibrils [284]. 

Enoxaparin is a low molecular weight heparin present in the intestinal mucosa of pigs. Enoxaparin reduced the Aβ load through the decreasing of β-secretase activity [285]. Also, enoxaparin has anti-inflammatory activity in vivo [286], improving the cognition of an AD mice model [287]. 

Morin, a natural flavonoid mainly found in *Maclura pomifera* (Raf.) C. K. Schneid. (Moraceae), *Maclura tinctoria* (L.) D. Don ex Steud. (Moraceae) and leaves of *Psidium guajava* L. (Myrtaceae), promoted the inhibition of β-secretase activity in vitro [190]. Besides, morin is able to reduce tau hyperphosphorylation in vivo [288]. 

Naringin is a flavonoid present in citrus fruits, namely in grapefruit. In vivo studies suggested that the antioxidant and anti-inflammatory activities of this compound improved the learning and memory of AD rats [289].

Vanillic acid is a phenolic acid extracted from the plant *Angelica sinensis* (Oliv.) Diels (apiaceae) with antioxidant and anti-inflammatory activities in vivo. As a result, an improvement in learning and memory of AD rats was observed [290].

Punicalagin is an ellagitannin found in the fruit peel of pomegranate (*Punica granatum* L. (Lythraceae)). In vivo studies suggest that punicalagin has potential as a nutritional preventive strategy in AD due to its anti-inflammatory activity. This natural product favors the anti-amylogenic route through the inhibition of β-secretase, reducing Aβ levels [291].

Piperine is a nitrogenous alkaloid found in fruits of the family *piperaceae*, including in *piper nigrum* L. and *piper longum* L. This compound has been used in traditional medicine to cure several diseases. In vivo trials reported that the reduction of lipid peroxidation can be linked with the neuroprotective effects of this compound [292], resulting in a significant improvement in memory of AD rat model [293].

Rhodosin is a flavonol extracted from the root of *Sedum roseum* (L.) Scop. (Crassulaceae) that improved the learning and memory injuries in an AD rat model due to its antioxidant activity [294]. 

### 3.2. Natural Extracts and Other Natural Products

Garlic (*Allium sativum* L., Amaryllidaceae) is frequently used in culinary and medicine. Several studies showed that the administration of aged garlic extract significantly improves the memory deficit by several pathways. In vitro studies demonstrated that aged garlic extract has antioxidant properties [295], inhibits Aβ fibril formation through the inhibition of Aβ aggregation [296] and it is able to defibrillate Aβ fibrils [296]. In addition, in vivo evidence showed that aged garlic extract has anti-inflammatory properties [297], increases the α-secretase activity and inhibits tau hyperphosphorylation [298].

Cinnamon (*Cinnamomum verum* J. Presl., Lauraceae) is one of the most used spices and has been traditionally applied in the treatment of some diseases and their symptoms. Cinnamon extract is found to inhibit in vitro tau aggregation and promote the disassembly of tau filaments [215]. Other in vitro studies suggested that the potential therapeutic effect of cinnamon against AD can also be due to its anti-inflammatory activity [299]. In vivo evidence showed that cinnamon extract has antioxidant activity [300], prevents Aβ oligomerization [301], reducing the Aβ level and correcting the cognitive impairment of transgenic mice [300]. 

Olive (*Olea europaea* L., *Oleaceae*) is the source of olive oil, one of the most important ingredients in the Mediterranean diet. In vivo studies showed that extra virgin olive oil ameliorated behavioural impairments. Also, the oil reduced the Aβ and phosphorylated tau levels [302]. This decrease can be due to the increase of Aβ clearance and APP modulation [303]. In vivo studies also proved its antioxidant activity, protecting against Aβ-induced cytotoxicity [304]. 

Walnut (*Juglans regia* L., Juglandaceae) is a dried fruit composed by fatty acids, vitamins, alpha tocopherol, and polyphenols, in particular ellagic acid. An in vitro study showed that walnut extract inhibited the Aβ fibril formation through the inhibition of Aβ fibrillation, and also defibrillated Aβ fibrils [305]. Additionally, in vivo studies demonstrated that walnut extract reduced the oxidative stress and neuroinflammation induced by Aβ in an AD mice model [306]. 

Grapes (*Vitis vinifera* L., Vitaceae) are composed by several polyphenols including catechin, epicatechin, epigallocatechin and epicatechin gallate. In vivo studies have revealed that grape seed extract increases the memory performance and reduces ROS production, thereby protecting the central nervous system [307]. An in vitro work revealed that grape seed extract blocks the Aβ fibril formation [308] through the inhibition of Aβ aggregation [309]. Therefore, the amount of amyloid plaques in the brain of AD mice was reduced. Besides, grape seed extract can attenuate the neuroinflammation in vivo [310]. In vivo works proved that the grape skin extract has antioxidant property [311] and inhibits the in vitro Aβ fibril formation [121,312].

Pomegranate (*Punica granatum* L., Lythraceae) is a fruit with a variety of antioxidant polyphenols. Pomegranate juice reduced the Aβ levels and amyloid plaques in an AD mouse model, improving spatial learning and cognitive performance [313]. Further in vivo analysis revealed that these results could be the product of the inhibition of γ-secretase activity [314]. In addition, in vivo studies demonstrated that pomegranate has anti-inflammatory [315] and antioxidant activities [316]. 

Skullcap (*Scutellaria baicalensis* Georgi, Lamiaceae) is a native American plant commonly used in traditional Chinese medicine. An in vivo study found that skullcap was able to protect hippocampal neurons against Aβ-induced damage through the attenuation of oxidative stress and neuroinflammation [317]. 

Strawberry (*Fragaria x ananassa* (Weston) Duchesne, Rosaceae) is known to contain high phenolic contents. In vivo studies showed that strawberries have anti-inflammatory [318] and antioxidant activities, protecting against oxidative stress [319].

Moringa (*Moringa oleifera* Lam., Moringaceae), an Asian and African plant, presents several nutrients, including β-carotene, vitamin C and E and phenols, including quercetin and kaempferol. In vivo studies showed that this plant improved the memory and learning due to its antioxidant activity [320].

## 4. Preclinical In Vitro Studies of Natural Compounds and Their Effects on AD

Besides the aforementioned natural compounds studied in human and animal studies, several other products have gained an increasing interest in scientific community for AD therapy. In fact, different compounds were tested in vitro and showed promising results. Some compounds proved to be efficient in preventing the formation of Aβ aggregates and disassembling Aβ fibrils, such as the case of tetracycline [321], methyl caffeate [322], retinol [171] and gou teng [323]. Also, other products demonstrated to be able to promote Aβ clearance, including withanolide A [242] and retinal [171].

The reduction of Aβ levels can occur through changes in the structure of Aβ aggregates induced by natural compounds such as piceatannol [324]. This product is also able to decrease Aβ levels through the activation of α-secretase. Withanolide A also promotes α-secretase expression and simultaneously inhibits β-secretase activity [242]. Other products proved to be inhibitors of β-secretase activity such as bastadin 9 [325], dictyodendrin [326], epicatechin gallate [327], gracilin [328], ianthellidone F [329], lamellarin O [329], neocoylin [330], tasiamide B [331], topsentinol K trisulfate [332] and xestosaprol [333].

Besides these mechanisms, natural compounds can prevent AD progression by other mechanisms. For example, yessotoxin [334], gambierol [335], gracilin [328], gymnodimine [336], palinurin [337] and schisandrone [338] reduced tau hyperphosphorylation. In addition, some compounds revealed to be able to suppress the oxidative stress by the scavenging of ROS and inflammatory response induced by Aβ, such as schisandrone [294], piceatannol [339], gracilin [340], sophocarpidine [294] and tetrahydroaplysulphurin-1 [340].

Despite the verified good outcomes, the study of some of these compounds was abandoned. For example, tetracycline was studied in 2001 but no more studies were reported for this compound. Also, for epicatechin gallate no studies were reported since 2003, and for retinal and retinol since 2004.

## 5. Discussion

Several bioactive compounds and natural extracts that were described herein to treat and prevent AD were revised and discussed. Until this date, most of the studied natural compounds are mainly derived from vegetable sources, with just a few molecules isolated from animals and marine organisms. Since AD is a multifactorial disorder, different therapeutic mechanisms were associated with these natural compounds. 

The approval process for a new compound to become clinically available is an extremely lengthy process, and it is divided into different phases. Before tests on humans, new compounds must be evaluated in preclinical studies. Several natural compounds proved to be promising for AD therapy in in vitro and in vivo studies, as discussed in this work. However, due to physiological differences between tested animals and humans, clinical trials are still necessary to validate the safety and efficacy of these compounds. Clinical studies are of outmost importance for the development of new therapeutic compounds, drugs and devices. Human studies allow to assess safety, tolerance and effective therapeutic doses for treating diseases. Some of the performed clinical trials described in this review did not show significant improvement in the delay or treatment of the symptoms. However, even if the trials do not exhibit positive outcomes, the obtained results can be still used to guide the scientists in the right path for drug discovery. Also, some of the conducted clinical trials with natural compounds for AD therapy, showed no conclusive results due to the limited size of samples. However, several compounds proved to be safe in human studies and were allowed to proceed to subsequent phases. To this date, homotaurine is the only compound that reached phase III of clinical trials for AD therapy.

**Figure 11:**
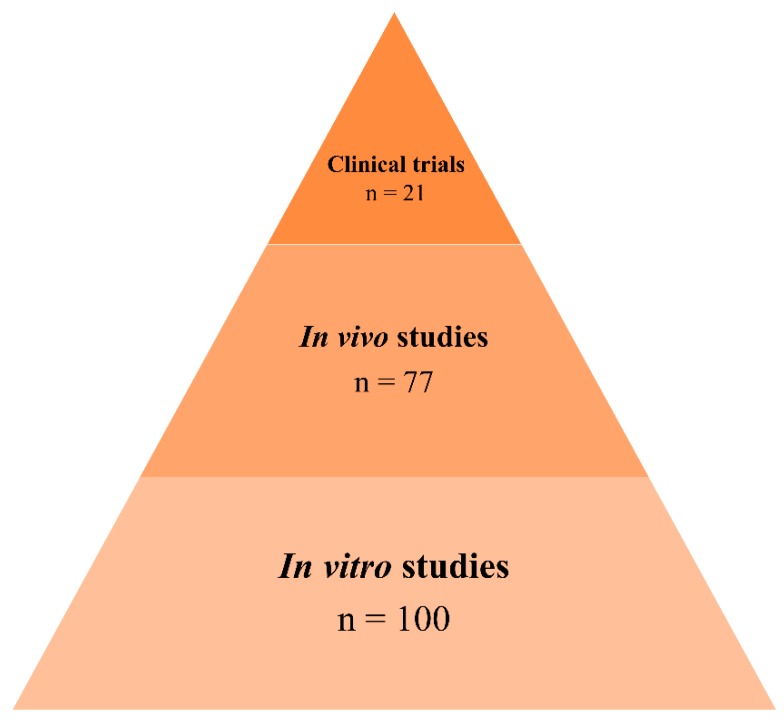
Number of natural products studied in different development phases.

Despite only a few natural products having been studied in clinical trials, numerous compounds proved to have beneficial properties in preclinical studies, as shown in Figure 11. Based on the works mentioned in this review, 21% of natural compounds achieved the clinical trials phase. However, it needs to be taken into account that since these types of products are commonly consumed in the daily life, it is easier to reach the phase I of clinical trials as they are supposed to be safe for humans. Unfortunately, not all these natural products demonstrated significant effects in the AD treatment. However, they could be used for AD prevention. In the next few years, it is expected that the number of natural compounds being studied in clinical trials for the prevention and treatment of AD will significantly increase. Since the enrichment of several food and beverages is a recent trend, fortification strategies using natural products could be a promising approach for AD prevention. In fact, some groups have studied the combination of different natural compounds. In 2009, a group started clinical trials for a beverage with supplementation of a mixture of natural compounds to be consumed by AD patients [341]. This supplement, commercially called Souvenaid^®^, demonstrates beneficial effects in the patients. This product is already commercially available in some countries being partially financially supported by the public health care systems.

Still, the neuroprotective effects of natural compounds depend of their ability to cross BBB. The low bioavailability of drugs and the difficulty to cross the BBB remains the major obstacles for the development of new therapies [342]. Drug delivery systems (DDS) targeting the brain seem to be a promising strategy to increase the bioavailability of compounds and the transport across the BBB [343]. DDS can protect the natural compounds from biological degradation and transport the molecules to the brain by masking their limiting physicochemical properties [344]. Thus, low doses of natural compounds are slowly released in the brain, increasing the efficiency of the therapeutic effects.

Among the studied natural compounds, only a small percentage have been encapsulated in DDS for brain targeting. Only the encapsulation of curcumin [345,346,347,348], epigallocatechin gallate [349,350], grape extracts [312], huperzine A [351], piperine [352], quercetin [353] and resveratrol [312] in functionalized DDS was reported in the literature. Therefore, some of these compounds seem to be the most promising for the AD treatment. One interesting approach could be the co-encapsulation in the same DDS of more than one natural compound with different therapeutic mechanisms, obtaining a synergistic effect. In the future, in addition to being necessary further studies to understand how natural compounds exert their therapeutic effects on AD, further experiments to target the drugs to the brain need to be performed.

## 6. Conclusions

AD is a disabling disorder with a major negative impact on our current society. At this moment, no drugs have been developed to prevent or treat AD. The existing molecules only aim to control the symptoms. With the increase of average life expectancy, it is fundamental to discover and develop new molecules able to prevent and treat AD. Several natural products have proven to be promising for AD therapy in clinical and preclinical studies. Clinical trials have shown that several compounds appear to be effective for AD therapy, whereas others have failed in human trials. Natural compounds in earlier phases of research need further studies to uncover their therapeutic potential for AD.

## Figures and Tables

**Table 1:** Bioactive compounds in clinical trials for AD therapy.

Bioactive Compound	Condition of Participants	Number of Subjects	Duration	Outcomes	Ref.
Vitamin D	Mild cognitive impairment	8	8 weeks	Reduction of Aβ level	[35]
	Mild cognitive impairment and early AD	48	20 months	Reduction of Aβ level; Improvement of cognitive functions	[36]
Vitamin D and memantine	Moderate AD	43	24 weeks	Improvement of cognitive functions	[37]
Vitamin D and antioxidants	Mild to moderate AD	78	16 weeks	Reduction of oxidative stress	[38]
Vitamin E and vitamin C	AD	20	1 month	Reduction of oxidative stress	[39]
Vitamin E and selegiline	Moderate AD	341	2 years	Delay of AD progression	[40]
Vitamin E and donepezil	Mild cognitive impairment	769	5 years	No effectiveness in delaying AD progression	[41]
Vitamin E and memantine	Mild to moderate AD	613	5 years	Delay of AD progression	[42]
Vitamin E and selenium	Healthy patients	3786	13 years	No prevention of dementia	[43]
Docosahexaenoic acid (DHA) and eicosapentaenoic acid	AD	204	12 months	Safe and well tolerated; No effectiveness in delaying cognitive decline	[44]
DHA	AD	295	18 months	No effectiveness in delaying cognitive decline	[45]
	Cognitive impairments	485	24 weeks	Improvement of cognitive functions	[46]
	Mild cognitive impairment	36	1 year	Safe and well tolerated; Improvement of memory	[47]
Homotaurine	Mild to moderate AD	1052	78 weeks	Improvement of cognitive functions	[48,49]
		58	3 months	No harmful effects on vital signs; Side effects	[50]
		10	4 weeks	Improvement of the central cholinergic transmission	[51]
Huperzine A	AD	103	8 weeks	Safe and well tolerated; Improvement of memory and behaviour	[52]
		60	60 days	Safe and well tolerated; Reduction of oxidative stress	[53]
	Mild to moderate AD	177	16 weeks	Safe and well tolerated; Improvement of cognitive functions	[54]
Bryostatin	AD	9	46 weeks	Safe and well tolerated: Improvement of cognitive functions	[55]
		150	12 weeks	Improvement of cognitive functions	[56]
Melatonin	AD	150	12 weeks	Improvement of memory	[57]
		14	22 to 35 months	Improvement of cognitive functions	[58]
	Mild cognitive impairment	50	9 to 18 months	Improvement of cognitive functions	[59]
	Mild to moderate AD	80	24 weeks	Safe; Improvement of cognitive functions	[60]
Resveratrol	Mild to moderate AD	119	52 weeks	Side effects; No effectiveness in reducing biomarkers levels	[61]
		39	1 year	Safe and well tolerated; No effectiveness in treat AD	[62]
Nicotine	AD	70	2 weeks	Improvement of perceptual and visual attentional deficits	[63]
		6	9 weeks	Safe; Improvement of learning	[64]
		8	10 weeks	Improvement of attentional performance	[65]
Curcumin	AD	34	6 months	Safe and well tolerated	[66]

**Table 2:** Natural extracts and other natural products in clinical trials for AD therapy.

Natural Extracts and Other Products	Condition of Participants	Number of Subjects	Duration	Outcomes	Ref.
Ginkgo biloba	Mild to moderate dementia	410	24 weeks	Safe; Improvement of neuropsychiatric symptoms	[67,68]
		410	24 weeks	Improvement of cognitive and functional functions	[69]
	AD or vascular dementia	404	24 weeks	Improvement of cognitive functions and functional abilities; Improvement of neuropsychiatric symptoms	[70]
	Mild cognitive impairment	160	24 weeks	Safe and well tolerated; Improvement of cognitive functions	[71]
Saffron	Mild to moderate AD	46	16 weeks	Safe; Improvement of cognitive functions and memory	[72]
Lemon balm	Mild to moderate AD	40	4 months	Improvement of cognition function and agitation	[73]
Green tea	Severe AD	30	2 months	Improvement of cognitive functions	[74]
Papaya	AD	20	6 months	Reduction of oxidative stress	[75]
Sage	Mild to moderate AD	20	4 months	Improvement of cognitive functions; No side effects except agitation	[76]
Coconut	AD	44	21 days	Improvement of cognitive functions	[77]
Apple	Moderate to severe AD	21	1 month	No improvement of cognitive functions; Improvement behavioural and psychotic symptoms; Reduction of anxiety, agitation and delusion	[78]
Blueberry	Early memory failures	9	12 weeks	Improvement of learning; Reduction of depressive symptoms	[79]
Colostrinin	AD	n. d.	15 weeks	Improvement of cognitive and daily functions	[80]

n. d.—The information was not provided by the authors.

## Abbreviations

Aβ	β-amyloid
ACH	Amyloid cascade hypothesis
AD	Alzheimer’s disease
APP	Amyloid precursor protein
BBB	Blood-brain barrier
DDS	Drug delivery systems
DHA	Docosahexanoic acid
EGCG	Epigallocatechin gallate
NDGA	Nordihydroguaiaretic acid
NFTs	Neurofibrillary tangles
PGG	1,2,3,4,6-Penta-*O*-galloyl-β-d-glucopyranose
ROS	Reactive oxygen species
